# 9-[(2-Chloro­benz­yl)amino]-5-(3,4,5-trimeth­oxy­phen­yl)-5,5a,8a,9-tetra­hydro­furo[3′,4′:6,7]naphtho­[2,3-*d*][1,3]dioxol-6(8*H*)-one

**DOI:** 10.1107/S1600536811018289

**Published:** 2011-05-25

**Authors:** Tie-Liang Zhu, Jie-Ru Jin, Hong Chen, Li-Ting Chen, Jing Liu

**Affiliations:** a The Affiliated Hospital of the Medical College of the Chinese People’s Armed Police Forces, Tianjin 300162, People’s Republic of China; b The General Unit of the Chinese People’s Armed Police Force Hospital in Tianjin, Tianjin 300252, People’s Republic of China; c Tianjin Key Laboratory for Biomarkers of Occupational and Environmental Hazards, Tianjin 300162, People’s Republic of China; d Room of Pharmacognosy, Medical College of the Chinese People’s Armed Police Forces, Tianjin 300162, People’s Republic of China

## Abstract

In the title compound, C_29_H_28_ClNO_7_, the tetra­hydro­furan ring and the six-membered ring fused to it both display envelope conformations. The dihedral angles between the plane of the benzene ring of the benzo[*d*][1,3]dioxole system and the planes of the other two benzene rings are 80.59 (3) and 63.60 (2)°.

## Related literature

For bond-length and angle data for similar structures, see: Feng *et al.* (2008 ▶); Zhang *et al.* (1994 ▶); Zuo *et al.* (2009 ▶).
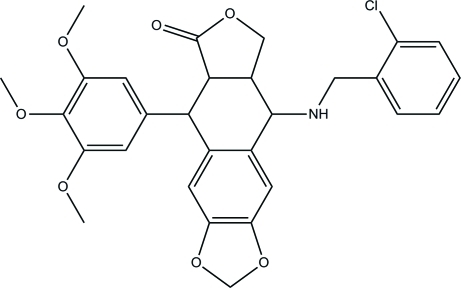


## Experimental

### 

#### Crystal data

C_29_H_28_ClNO_7_
*M* *_r_* = 537.97
Orthorhombic, 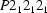

*a* = 10.0971 (14) Å
*b* = 15.264 (2) Å
*c* = 16.220 (2) Å
*V* = 2499.9 (6) Å^3^
*Z* = 4
Mo *K*α radiation
μ = 0.20 mm^−1^
*T* = 113 K
0.20 × 0.18 × 0.12 mm

#### Data collection

Rigaku Saturn CCD area-detector diffractometer
Absorption correction: multi-scan (*CrystalClear*; Rigaku, 2007 ▶) *T*_min_ = 0.960, *T*_max_ = 0.976
26247 measured reflections
5968 independent reflections
5580 reflections with *I* > 2σ(*I*)
*R*_int_ = 0.044

#### Refinement

*R*[*F* ^2^ > 2σ(*F* ^2^)] = 0.031
*wR*(*F* ^2^) = 0.066
*S* = 1.03
5968 reflections
350 parameters
H atoms treated by a mixture of independent and constrained refinement
Δρ_max_ = 0.18 e Å^−3^
Δρ_min_ = −0.25 e Å^−3^
Absolute structure: Flack (1983), 2615 Friedel pairs
Flack parameter: 0.00 (4)

### 

Data collection: *CrystalClear* (Rigaku, 2007 ▶); cell refinement: *CrystalClear*; data reduction: *CrystalClear*; program(s) used to solve structure: *SHELXS97* (Sheldrick, 2008 ▶); program(s) used to refine structure: *SHELXL97* (Sheldrick, 2008 ▶); molecular graphics: *SHELXTL* (Sheldrick, 2008 ▶); software used to prepare material for publication: *SHELXL97*.

## Supplementary Material

Crystal structure: contains datablocks I, global. DOI: 10.1107/S1600536811018289/hg5035sup1.cif

Structure factors: contains datablocks I. DOI: 10.1107/S1600536811018289/hg5035Isup2.hkl

Supplementary material file. DOI: 10.1107/S1600536811018289/hg5035Isup3.cml

Additional supplementary materials:  crystallographic information; 3D view; checkCIF report

## Figures and Tables

**Table 1:** Hydrogen-bond geometry (Å, °)

*D*—H⋯*A*	*D*—H	H⋯*A*	*D*⋯*A*	*D*—H⋯*A*
C26—H26⋯O7^i^	0.95	2.56	3.2130 (18)	126

Symmetry code: (i) 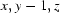.

**Fig. 1.**
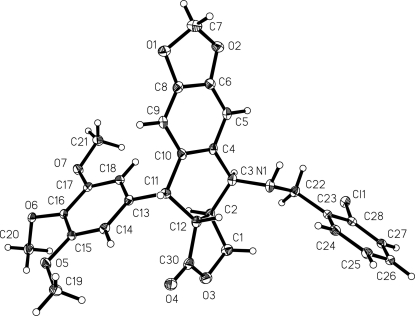
View of the title compound, with displacement ellipsoids drawn at the 40% probability level.

## supplementary crystallographic information

### Comment

Podophyllotoxin and their derivatives are well known as substances with anti-cancer activity. In recent years, our study are paying attention to synthesize different kinds of Podophyllotoxin compounds and aim at the discovery of new derivatives with improved bioactivities. In this paper, we reported the crystal structure of title compound.

In title compound, C_29_H_28_ClNO_7_, bond lengths and angles are normal and in good agreement with those reported previously (Feng *et al.*, 2008; Zhang, *et al.*, 1994; Zuo, *et al.*, 2009). The tetrahydrofuran ring (C1/C2/C12/C30/O3) and the six-membered ring (C2—C4/C10—C12)fused to it both display envelope conformations. The dihedral angles between the benzene ring (C4—C10) of the benzo[*d*]-[1,3]dioxole and the other two benzene ring (C13—C18 and C23—C28) are 80.59 (3) and 63.60 (2)°, respectively. There are weaker C—H···O intermolecular interactions, which stabilized the structure (Table 1).

### Experimental

The target compound was synthesized by two steps. 2-chlorobenzaldehyde, 4β-amino podophyllotoxin, two drops of acetic acid in 95% ethanol was stirred for 6 h. Appropriate amount of NaBH_4_ was added into the reaction mixture to stirred for 1 h at 273 K. Then add 5% HCl to end off the reaction, the reaction mixture was concentrated *in vacuo*. Add saturated NaHCO_3_ to adjust PH>7. The reaction mixture was extracted with CH_2_Cl_2_ and dried over MgSO_4_ and concentrated *in vacuo*. The residue was resolved in a methanol solution and slow evaporation over two weeks at room temperature gave transparent crystals suitable for X-ray analysis.

### Refinement

All C H atoms were found on difference maps, with C—H = 0.95–1.00 Å and included in the final cycles of refinement using a riding model, with *U*_iso_(H) = 1.2*U*_eq_(C) for aryl and methylene H atoms and 1.5*U*_eq_(C) for the methyl H atoms.  H atoms bonded N were refined freely with N—H = 0.96 (2) Å.

### Crystal data

**Table:**

C_29_H_28_ClNO_7_	*F*(000) = 1128
*M**_r_* = 537.97	*D*_x_ = 1.429 Mg m^−^^3^
Orthorhombic, *P*2_1_2_1_2_1_	Mo *K*α radiation, λ = 0.71073 Å
Hall symbol: P 2ac 2ab	Cell parameters from 8535 reflections
*a* = 10.0971 (14) Å	θ = 1.3–27.9°
*b* = 15.264 (2) Å	µ = 0.20 mm^−^^1^
*c* = 16.220 (2) Å	*T* = 113 K
*V* = 2499.9 (6) Å^3^	Prism, colorless
*Z* = 4	0.20 × 0.18 × 0.12 mm

### Data collection

**Table:**

Rigaku Saturn CCD area-detector diffractometer	5968 independent reflections
Radiation source: rotating anode	5580 reflections with *I* > 2σ(*I*)
multilayer	*R*_int_ = 0.044
Detector resolution: 14.63 pixels mm^-1^	θ_max_ = 27.9°, θ_min_ = 1.8°
ω and φ scans	*h* = −13→13
Absorption correction: multi-scan (*CrystalClear*; Rigaku, 2007)	*k* = −20→20
*T*_min_ = 0.960, *T*_max_ = 0.976	*l* = −20→21
26247 measured reflections	

### Refinement

**Table:**

Refinement on *F*^2^	Secondary atom site location: difference Fourier map
Least-squares matrix: full	Hydrogen site location: inferred from neighbouring sites
*R*[*F*^2^ > 2σ(*F*^2^)] = 0.031	H atoms treated by a mixture of independent and constrained refinement
*wR*(*F*^2^) = 0.066	*w* = 1/[σ^2^(*F*_o_^2^) + (0.0324*P*)^2^] where *P* = (*F*_o_^2^ + 2*F*_c_^2^)/3
*S* = 1.03	(Δ/σ)_max_ = 0.001
5968 reflections	Δρ_max_ = 0.18 e Å^−^^3^
350 parameters	Δρ_min_ = −0.25 e Å^−^^3^
0 restraints	Absolute structure: Flack (1983), 2615 Friedel pairs
Primary atom site location: structure-invariant direct methods	Flack parameter: 0.00 (4)

### Special details

**Table:**

Geometry. All e.s.d.'s (except the e.s.d. in the dihedral angle between two l.s. planes) are estimated using the full covariance matrix. The cell e.s.d.'s are taken into account individually in the estimation of e.s.d.'s in distances, angles and torsion angles; correlations between e.s.d.'s in cell parameters are only used when they are defined by crystal symmetry. An approximate (isotropic) treatment of cell e.s.d.'s is used for estimating e.s.d.'s involving l.s. planes.
Refinement. Refinement of *F*^2^ against ALL reflections. The weighted *R*-factor *wR* and goodness of fit *S* are based on *F*^2^, conventional *R*-factors *R* are based on *F*, with *F* set to zero for negative *F*^2^. The threshold expression of *F*^2^ > σ(*F*^2^) is used only for calculating *R*-factors(gt) *etc*. and is not relevant to the choice of reflections for refinement. *R*-factors based on *F*^2^ are statistically about twice as large as those based on *F*, and *R*- factors based on ALL data will be even larger.

### Fractional atomic coordinates and isotropic or equivalent isotropic displacement parameters (Å^2^)

**Table:**

	*x*	*y*	*z*	*U*_iso_*/*U*_eq_	
Cl1	−0.19083 (4)	−0.19218 (2)	0.81504 (2)	0.02120 (9)	
O1	0.56079 (11)	0.23021 (7)	0.72078 (6)	0.0247 (3)	
O2	0.41961 (12)	0.14072 (7)	0.64668 (6)	0.0240 (3)	
O3	0.09055 (11)	0.06096 (7)	1.11792 (6)	0.0253 (3)	
O4	0.28209 (11)	0.10425 (6)	1.17321 (6)	0.0261 (3)	
O5	0.31178 (11)	0.40151 (6)	1.20050 (6)	0.0209 (2)	
O6	0.18781 (11)	0.51133 (6)	1.09222 (6)	0.0189 (2)	
O7	0.12116 (11)	0.45705 (6)	0.94280 (6)	0.0209 (2)	
N1	0.16797 (13)	−0.03792 (7)	0.88773 (8)	0.0199 (3)	
C1	0.03959 (16)	0.03821 (10)	1.03598 (9)	0.0229 (3)	
H1A	−0.0550	0.0545	1.0307	0.027*	
H1B	0.0491	−0.0253	1.0253	0.027*	
C2	0.12467 (15)	0.09136 (9)	0.97679 (9)	0.0178 (3)	
H2	0.0883	0.1522	0.9740	0.021*	
C3	0.14712 (15)	0.05866 (8)	0.88890 (9)	0.0180 (3)	
H3	0.0667	0.0725	0.8554	0.022*	
C4	0.26551 (15)	0.10614 (8)	0.85124 (9)	0.0162 (3)	
C5	0.28349 (15)	0.09545 (9)	0.76558 (9)	0.0193 (3)	
H5	0.2256	0.0594	0.7343	0.023*	
C6	0.38597 (16)	0.13821 (9)	0.72912 (9)	0.0189 (3)	
C7	0.54559 (17)	0.18360 (12)	0.64454 (10)	0.0287 (4)	
H7A	0.5496	0.2248	0.5975	0.034*	
H7B	0.6174	0.1399	0.6382	0.034*	
C8	0.47041 (15)	0.19194 (10)	0.77350 (9)	0.0185 (3)	
C9	0.45689 (15)	0.20311 (9)	0.85659 (9)	0.0180 (3)	
H9	0.5156	0.2400	0.8865	0.022*	
C10	0.35316 (15)	0.15831 (8)	0.89679 (9)	0.0163 (3)	
C11	0.34335 (15)	0.16871 (8)	0.99015 (8)	0.0161 (3)	
H11	0.4342	0.1599	1.0133	0.019*	
C12	0.25620 (15)	0.09411 (9)	1.02231 (9)	0.0179 (3)	
H12	0.3033	0.0382	1.0093	0.021*	
C13	0.29684 (15)	0.25985 (8)	1.01666 (8)	0.0158 (3)	
C14	0.32622 (15)	0.28769 (8)	1.09600 (9)	0.0168 (3)	
H14	0.3742	0.2503	1.1320	0.020*	
C15	0.28577 (15)	0.37031 (9)	1.12340 (8)	0.0158 (3)	
C16	0.21789 (15)	0.42648 (8)	1.07030 (8)	0.0158 (3)	
C17	0.18852 (15)	0.39801 (8)	0.99061 (8)	0.0157 (3)	
C18	0.22750 (14)	0.31519 (9)	0.96379 (9)	0.0165 (3)	
H18	0.2068	0.2965	0.9094	0.020*	
C19	0.37066 (17)	0.34058 (10)	1.25669 (9)	0.0248 (4)	
H19A	0.3180	0.2867	1.2580	0.037*	
H19B	0.3734	0.3664	1.3120	0.037*	
H19C	0.4609	0.3270	1.2386	0.037*	
C20	0.08614 (17)	0.52134 (10)	1.15354 (10)	0.0233 (4)	
H20A	0.0070	0.4889	1.1365	0.035*	
H20B	0.0642	0.5836	1.1596	0.035*	
H20C	0.1179	0.4984	1.2064	0.035*	
C21	0.10712 (18)	0.43615 (10)	0.85726 (9)	0.0254 (4)	
H21A	0.1949	0.4285	0.8325	0.038*	
H21B	0.0604	0.4838	0.8291	0.038*	
H21C	0.0564	0.3818	0.8515	0.038*	
C22	0.04768 (15)	−0.08553 (9)	0.86421 (9)	0.0193 (3)	
H22A	0.0434	−0.0891	0.8033	0.023*	
H22B	−0.0305	−0.0519	0.8832	0.023*	
C23	0.04024 (15)	−0.17743 (9)	0.89942 (8)	0.0157 (3)	
C24	0.13828 (16)	−0.21272 (9)	0.94947 (9)	0.0196 (3)	
H24	0.2148	−0.1789	0.9617	0.024*	
C25	0.12665 (16)	−0.29669 (9)	0.98209 (9)	0.0218 (3)	
H25	0.1958	−0.3201	1.0151	0.026*	
C26	0.01461 (16)	−0.34623 (10)	0.96657 (9)	0.0206 (3)	
H26	0.0055	−0.4028	0.9904	0.025*	
C27	−0.08436 (15)	−0.31306 (9)	0.91619 (8)	0.0184 (3)	
H27	−0.1614	−0.3467	0.9049	0.022*	
C28	−0.06935 (15)	−0.23039 (9)	0.88264 (8)	0.0165 (3)	
C30	0.21676 (16)	0.08957 (9)	1.11209 (9)	0.0211 (3)	
H1	0.2339 (18)	−0.0558 (10)	0.8486 (10)	0.029 (5)*	

### Atomic displacement parameters (Å^2^)

**Table:**

	*U*^11^	*U*^22^	*U*^33^	*U*^12^	*U*^13^	*U*^23^
Cl1	0.01945 (19)	0.02013 (16)	0.02402 (18)	−0.00103 (16)	−0.00483 (16)	0.00179 (14)
O1	0.0245 (6)	0.0268 (6)	0.0227 (6)	−0.0046 (5)	0.0060 (5)	−0.0004 (4)
O2	0.0276 (7)	0.0272 (6)	0.0172 (5)	−0.0013 (5)	0.0006 (5)	−0.0011 (4)
O3	0.0264 (7)	0.0266 (6)	0.0229 (6)	−0.0033 (5)	0.0008 (5)	0.0018 (5)
O4	0.0340 (7)	0.0244 (5)	0.0200 (6)	−0.0020 (5)	−0.0058 (5)	0.0028 (4)
O5	0.0264 (6)	0.0203 (5)	0.0160 (5)	−0.0005 (5)	−0.0052 (5)	−0.0020 (4)
O6	0.0253 (6)	0.0112 (4)	0.0203 (5)	−0.0021 (5)	0.0045 (5)	−0.0013 (4)
O7	0.0305 (6)	0.0163 (5)	0.0159 (5)	0.0054 (5)	−0.0063 (5)	0.0013 (4)
N1	0.0177 (7)	0.0138 (6)	0.0282 (7)	0.0003 (5)	−0.0035 (6)	−0.0016 (5)
C1	0.0202 (9)	0.0249 (8)	0.0234 (8)	−0.0020 (7)	−0.0009 (7)	0.0017 (6)
C2	0.0168 (8)	0.0154 (7)	0.0213 (7)	0.0017 (6)	−0.0017 (6)	0.0016 (6)
C3	0.0163 (8)	0.0147 (7)	0.0230 (8)	0.0008 (6)	−0.0051 (6)	−0.0003 (6)
C4	0.0178 (8)	0.0104 (6)	0.0203 (7)	0.0030 (6)	−0.0027 (6)	0.0012 (5)
C5	0.0213 (8)	0.0140 (6)	0.0227 (8)	0.0009 (6)	−0.0054 (6)	−0.0020 (6)
C6	0.0232 (9)	0.0165 (7)	0.0171 (8)	0.0050 (6)	−0.0008 (6)	0.0017 (6)
C7	0.0255 (9)	0.0387 (10)	0.0219 (8)	−0.0006 (8)	0.0017 (7)	0.0007 (7)
C8	0.0170 (8)	0.0152 (7)	0.0232 (8)	0.0026 (7)	0.0003 (6)	0.0023 (6)
C9	0.0176 (8)	0.0142 (7)	0.0221 (8)	0.0001 (6)	−0.0023 (6)	−0.0031 (6)
C10	0.0179 (8)	0.0126 (6)	0.0182 (7)	0.0059 (6)	−0.0010 (6)	−0.0014 (5)
C11	0.0169 (8)	0.0164 (7)	0.0151 (7)	0.0013 (6)	−0.0033 (6)	0.0010 (5)
C12	0.0184 (8)	0.0139 (6)	0.0214 (8)	0.0027 (6)	−0.0028 (6)	0.0012 (6)
C13	0.0142 (8)	0.0153 (7)	0.0179 (7)	−0.0017 (6)	0.0000 (7)	0.0002 (5)
C14	0.0157 (8)	0.0161 (7)	0.0187 (7)	0.0005 (6)	−0.0041 (6)	0.0036 (5)
C15	0.0154 (8)	0.0179 (6)	0.0140 (7)	−0.0047 (6)	−0.0006 (6)	−0.0013 (5)
C16	0.0181 (8)	0.0125 (6)	0.0169 (7)	−0.0027 (6)	0.0024 (6)	−0.0017 (5)
C17	0.0148 (7)	0.0146 (6)	0.0178 (7)	−0.0023 (6)	0.0002 (6)	0.0030 (5)
C18	0.0174 (7)	0.0169 (7)	0.0152 (7)	−0.0020 (6)	0.0010 (6)	−0.0006 (6)
C19	0.0292 (9)	0.0276 (8)	0.0175 (8)	−0.0019 (7)	−0.0070 (7)	0.0028 (6)
C20	0.0239 (9)	0.0189 (8)	0.0272 (9)	−0.0012 (6)	0.0056 (7)	−0.0033 (6)
C21	0.0386 (10)	0.0215 (8)	0.0160 (8)	−0.0003 (7)	−0.0056 (7)	0.0025 (6)
C22	0.0196 (8)	0.0172 (7)	0.0213 (8)	−0.0031 (6)	−0.0033 (6)	0.0018 (6)
C23	0.0182 (8)	0.0151 (7)	0.0138 (7)	0.0006 (6)	0.0017 (6)	−0.0017 (5)
C24	0.0202 (8)	0.0212 (8)	0.0174 (7)	−0.0011 (6)	−0.0004 (6)	−0.0022 (6)
C25	0.0274 (9)	0.0211 (8)	0.0169 (7)	0.0059 (7)	−0.0040 (7)	0.0001 (6)
C26	0.0284 (9)	0.0158 (7)	0.0174 (7)	0.0022 (6)	0.0046 (7)	0.0003 (6)
C27	0.0200 (8)	0.0183 (7)	0.0169 (7)	−0.0029 (7)	0.0044 (6)	−0.0025 (6)
C28	0.0168 (8)	0.0189 (7)	0.0139 (7)	0.0018 (6)	0.0002 (6)	−0.0018 (6)
C30	0.0246 (9)	0.0143 (7)	0.0245 (8)	0.0018 (6)	−0.0008 (7)	0.0032 (6)

### Geometric parameters (Å, °)

**Table:**

Cl1—C28	1.7455 (15)	C11—C13	1.5301 (19)
O1—C8	1.3803 (18)	C11—C12	1.531 (2)
O1—C7	1.4348 (18)	C11—H11	1.0000
O2—C6	1.3802 (17)	C12—C30	1.511 (2)
O2—C7	1.431 (2)	C12—H12	1.0000
O3—C30	1.3504 (19)	C13—C14	1.3874 (19)
O3—C1	1.4669 (18)	C13—C18	1.3925 (19)
O4—C30	1.2116 (18)	C14—C15	1.3981 (19)
O5—C15	1.3637 (16)	C14—H14	0.9500
O5—C19	1.4315 (17)	C15—C16	1.395 (2)
O6—C16	1.3771 (16)	C16—C17	1.3956 (19)
O6—C20	1.4374 (18)	C17—C18	1.3937 (19)
O7—C17	1.3697 (17)	C18—H18	0.9500
O7—C21	1.4307 (18)	C19—H19A	0.9800
N1—C22	1.4659 (19)	C19—H19B	0.9800
N1—C3	1.4892 (17)	C19—H19C	0.9800
N1—H1	0.959 (17)	C20—H20A	0.9800
C1—C2	1.522 (2)	C20—H20B	0.9800
C1—H1A	0.9900	C20—H20C	0.9800
C1—H1B	0.9900	C21—H21A	0.9800
C2—C12	1.520 (2)	C21—H21B	0.9800
C2—C3	1.527 (2)	C21—H21C	0.9800
C2—H2	1.0000	C22—C23	1.5164 (19)
C3—C4	1.526 (2)	C22—H22A	0.9900
C3—H3	1.0000	C22—H22B	0.9900
C4—C10	1.401 (2)	C23—C24	1.389 (2)
C4—C5	1.411 (2)	C23—C28	1.397 (2)
C5—C6	1.359 (2)	C24—C25	1.392 (2)
C5—H5	0.9500	C24—H24	0.9500
C6—C8	1.385 (2)	C25—C26	1.384 (2)
C7—H7A	0.9900	C25—H25	0.9500
C7—H7B	0.9900	C26—C27	1.387 (2)
C8—C9	1.3654 (19)	C26—H26	0.9500
C9—C10	1.411 (2)	C27—C28	1.383 (2)
C9—H9	0.9500	C27—H27	0.9500
C10—C11	1.526 (2)		
			
C8—O1—C7	104.68 (11)	C14—C13—C18	119.54 (12)
C6—O2—C7	104.77 (12)	C14—C13—C11	118.26 (12)
C30—O3—C1	110.13 (12)	C18—C13—C11	122.19 (12)
C15—O5—C19	115.91 (11)	C13—C14—C15	120.58 (13)
C16—O6—C20	115.86 (10)	C13—C14—H14	119.7
C17—O7—C21	116.85 (11)	C15—C14—H14	119.7
C22—N1—C3	112.14 (11)	O5—C15—C16	116.50 (12)
C22—N1—H1	105.2 (10)	O5—C15—C14	123.39 (12)
C3—N1—H1	112.8 (10)	C16—C15—C14	120.10 (13)
O3—C1—C2	104.31 (12)	O6—C16—C15	121.80 (12)
O3—C1—H1A	110.9	O6—C16—C17	119.02 (12)
C2—C1—H1A	110.9	C15—C16—C17	119.00 (12)
O3—C1—H1B	110.9	O7—C17—C18	124.07 (13)
C2—C1—H1B	110.9	O7—C17—C16	115.16 (12)
H1A—C1—H1B	108.9	C18—C17—C16	120.77 (13)
C12—C2—C1	101.62 (11)	C13—C18—C17	120.00 (13)
C12—C2—C3	109.44 (12)	C13—C18—H18	120.0
C1—C2—C3	119.88 (12)	C17—C18—H18	120.0
C12—C2—H2	108.4	O5—C19—H19A	109.5
C1—C2—H2	108.4	O5—C19—H19B	109.5
C3—C2—H2	108.4	H19A—C19—H19B	109.5
N1—C3—C4	110.76 (12)	O5—C19—H19C	109.5
N1—C3—C2	110.88 (12)	H19A—C19—H19C	109.5
C4—C3—C2	109.56 (11)	H19B—C19—H19C	109.5
N1—C3—H3	108.5	O6—C20—H20A	109.5
C4—C3—H3	108.5	O6—C20—H20B	109.5
C2—C3—H3	108.5	H20A—C20—H20B	109.5
C10—C4—C5	120.25 (14)	O6—C20—H20C	109.5
C10—C4—C3	123.63 (13)	H20A—C20—H20C	109.5
C5—C4—C3	116.12 (13)	H20B—C20—H20C	109.5
C6—C5—C4	118.11 (14)	O7—C21—H21A	109.5
C6—C5—H5	120.9	O7—C21—H21B	109.5
C4—C5—H5	120.9	H21A—C21—H21B	109.5
C5—C6—O2	128.50 (14)	O7—C21—H21C	109.5
C5—C6—C8	121.80 (13)	H21A—C21—H21C	109.5
O2—C6—C8	109.61 (13)	H21B—C21—H21C	109.5
O2—C7—O1	107.52 (12)	N1—C22—C23	113.68 (12)
O2—C7—H7A	110.2	N1—C22—H22A	108.8
O1—C7—H7A	110.2	C23—C22—H22A	108.8
O2—C7—H7B	110.2	N1—C22—H22B	108.8
O1—C7—H7B	110.2	C23—C22—H22B	108.8
H7A—C7—H7B	108.5	H22A—C22—H22B	107.7
C9—C8—O1	128.67 (14)	C24—C23—C28	117.00 (13)
C9—C8—C6	121.71 (14)	C24—C23—C22	122.93 (13)
O1—C8—C6	109.61 (12)	C28—C23—C22	120.07 (13)
C8—C9—C10	118.03 (14)	C23—C24—C25	121.28 (14)
C8—C9—H9	121.0	C23—C24—H24	119.4
C10—C9—H9	121.0	C25—C24—H24	119.4
C4—C10—C9	120.05 (13)	C26—C25—C24	120.20 (14)
C4—C10—C11	122.78 (13)	C26—C25—H25	119.9
C9—C10—C11	117.17 (13)	C24—C25—H25	119.9
C10—C11—C13	113.17 (11)	C25—C26—C27	119.80 (14)
C10—C11—C12	107.35 (11)	C25—C26—H26	120.1
C13—C11—C12	113.84 (12)	C27—C26—H26	120.1
C10—C11—H11	107.4	C28—C27—C26	119.09 (14)
C13—C11—H11	107.4	C28—C27—H27	120.5
C12—C11—H11	107.4	C26—C27—H27	120.5
C30—C12—C2	103.68 (12)	C27—C28—C23	122.56 (14)
C30—C12—C11	120.93 (12)	C27—C28—Cl1	118.38 (12)
C2—C12—C11	110.93 (11)	C23—C28—Cl1	119.05 (11)
C30—C12—H12	106.8	O4—C30—O3	121.09 (14)
C2—C12—H12	106.8	O4—C30—C12	129.53 (15)
C11—C12—H12	106.8	O3—C30—C12	109.32 (13)
			
C30—O3—C1—C2	−23.55 (15)	C10—C11—C13—C14	−158.29 (13)
O3—C1—C2—C12	32.23 (14)	C12—C11—C13—C14	78.78 (17)
O3—C1—C2—C3	152.89 (12)	C10—C11—C13—C18	21.3 (2)
C22—N1—C3—C4	−138.33 (13)	C12—C11—C13—C18	−101.68 (15)
C22—N1—C3—C2	99.82 (14)	C18—C13—C14—C15	0.6 (2)
C12—C2—C3—N1	76.31 (15)	C11—C13—C14—C15	−179.85 (13)
C1—C2—C3—N1	−40.37 (18)	C19—O5—C15—C16	174.55 (13)
C12—C2—C3—C4	−46.24 (14)	C19—O5—C15—C14	−7.0 (2)
C1—C2—C3—C4	−162.92 (13)	C13—C14—C15—O5	−179.86 (13)
N1—C3—C4—C10	−111.37 (14)	C13—C14—C15—C16	−1.5 (2)
C2—C3—C4—C10	11.25 (18)	C20—O6—C16—C15	−71.65 (18)
N1—C3—C4—C5	68.97 (15)	C20—O6—C16—C17	113.25 (15)
C2—C3—C4—C5	−168.41 (12)	O5—C15—C16—O6	4.9 (2)
C10—C4—C5—C6	−1.1 (2)	C14—C15—C16—O6	−173.55 (13)
C3—C4—C5—C6	178.52 (12)	O5—C15—C16—C17	−179.98 (12)
C4—C5—C6—O2	−177.04 (14)	C14—C15—C16—C17	1.5 (2)
C4—C5—C6—C8	−0.8 (2)	C21—O7—C17—C18	−9.0 (2)
C7—O2—C6—C5	−171.48 (15)	C21—O7—C17—C16	170.60 (13)
C7—O2—C6—C8	11.94 (16)	O6—C16—C17—O7	−5.1 (2)
C6—O2—C7—O1	−19.05 (16)	C15—C16—C17—O7	179.68 (13)
C8—O1—C7—O2	18.94 (16)	O6—C16—C17—C18	174.50 (13)
C7—O1—C8—C9	169.91 (16)	C15—C16—C17—C18	−0.7 (2)
C7—O1—C8—C6	−11.63 (15)	C14—C13—C18—C17	0.2 (2)
C5—C6—C8—C9	1.6 (2)	C11—C13—C18—C17	−179.31 (13)
O2—C6—C8—C9	178.41 (13)	O7—C17—C18—C13	179.40 (13)
C5—C6—C8—O1	−177.03 (13)	C16—C17—C18—C13	−0.1 (2)
O2—C6—C8—O1	−0.17 (16)	C3—N1—C22—C23	−152.51 (12)
O1—C8—C9—C10	178.05 (13)	N1—C22—C23—C24	0.2 (2)
C6—C8—C9—C10	−0.2 (2)	N1—C22—C23—C28	179.56 (13)
C5—C4—C10—C9	2.4 (2)	C28—C23—C24—C25	−0.7 (2)
C3—C4—C10—C9	−177.21 (13)	C22—C23—C24—C25	178.61 (14)
C5—C4—C10—C11	−176.76 (13)	C23—C24—C25—C26	−1.5 (2)
C3—C4—C10—C11	3.6 (2)	C24—C25—C26—C27	2.1 (2)
C8—C9—C10—C4	−1.7 (2)	C25—C26—C27—C28	−0.4 (2)
C8—C9—C10—C11	177.53 (13)	C26—C27—C28—C23	−2.0 (2)
C4—C10—C11—C13	−109.51 (15)	C26—C27—C28—Cl1	176.65 (11)
C9—C10—C11—C13	71.28 (17)	C24—C23—C28—C27	2.5 (2)
C4—C10—C11—C12	16.95 (18)	C22—C23—C28—C27	−176.88 (13)
C9—C10—C11—C12	−162.27 (12)	C24—C23—C28—Cl1	−176.10 (11)
C1—C2—C12—C30	−29.50 (14)	C22—C23—C28—Cl1	4.52 (18)
C3—C2—C12—C30	−157.22 (11)	C1—O3—C30—O4	−173.15 (13)
C1—C2—C12—C11	−160.76 (12)	C1—O3—C30—C12	4.19 (15)
C3—C2—C12—C11	71.52 (14)	C2—C12—C30—O4	−166.14 (15)
C10—C11—C12—C30	−174.77 (13)	C11—C12—C30—O4	−41.1 (2)
C13—C11—C12—C30	−48.71 (18)	C2—C12—C30—O3	16.81 (14)
C10—C11—C12—C2	−53.14 (15)	C11—C12—C30—O3	141.88 (13)
C13—C11—C12—C2	72.92 (15)		

### Hydrogen-bond geometry (Å, °)

**Table:**

*D*—H···*A*	*D*—H	H···*A*	*D*···*A*	*D*—H···*A*
C26—H26···O7^i^	0.95	2.56	3.2130 (18)	126.

Symmetry codes: (i) *x*, *y*−1, *z*.
